# Evaluation of two methods of estimating larval habitat productivity in western Kenya highlands

**DOI:** 10.1186/1756-3305-4-110

**Published:** 2011-06-17

**Authors:** Eliningaya J Kweka, Guofa Zhou, Ming-Chieh Lee, Thomas M Gilbreath, Franklin Mosha, Stephen Munga, Andrew K Githeko, Guiyun Yan

**Affiliations:** 1 Centre for Global Health Research, Kenya Medical Research Institute, P. O. Box 1578, Kisumu 40100, Kenya; 2 Kilimanjaro Christian Medical College of Tumaini University, P. O. Box 2240, Moshi, Tanzania; 3 Program in Public Health, University of California, Irvine, CA 92697, USA

## Abstract

**Background:**

Malaria vector intervention and control programs require reliable and accurate information about vector abundance and their seasonal distribution. The availability of reliable information on the spatial and temporal productivity of larval vector habitats can improve targeting of larval control interventions and our understanding of local malaria transmission and epidemics. The main objective of this study was to evaluate two methods of estimating larval habitat productivity in the western Kenyan highlands, the aerial sampler and the emergence trap.

**Methods:**

The study was conducted during the dry and rainy seasons in 2008, 2009 and 2010. Aerial samplers and emergence traps were set up for sixty days in each season in three habitat types: drainage ditches, natural swamps, and abandoned goldmines. Aerial samplers and emergence traps were set up in eleven places in each habitat type. The success of each in estimating habitat productivity was assessed according to method, habitat type, and season. The effect of other factors including algae cover, grass cover, habitat depth and width, and habitat water volume on species productivity was analysed using stepwise logistic regression

**Results:**

Habitat productivity estimates obtained by the two sampling methods differed significantly for all species except for *An*. *implexus*. For for *An*. *gambiae *s.l. and *An*. *funestus*, aerial samplers performed better, 21.5 and 14.6 folds, than emergence trap respectively, while the emergence trap was shown to be more efficient for culicine species. Seasonality had a significant influence on the productivity of all species monitored. Dry season was most productive season. Overall, drainage ditches had significantly higher productivity in all seasons compared to other habitat types. Algae cover, debris, chlorophyll-a, and habitat depth and size had significant influence with respect to species.

**Conclusion:**

These findings suggest that the aerial sampler is the better of the two methods for estimating the productivity of *An*. *gambiae *s.l. and *An*. *funestus *in the western Kenya highlands and possibly other malaria endemic parts of Africa. This method has proven to be a useful tool for monitoring malaria vector populations and for control program design, and provides useful means for determining the most suitable sites for targeted interventions.

## Background

Although recent studies [1-3], have demonstrated that malaria disease burden is on the decline in several sub-Saharan African countries, the disease still remains a major public health problem. This reduction in the disease burden has parsimoniously been attributed to recent scale-up of control tools in some parts of Africa [2]. To design effective vector control programmes, national malaria control programmes in Africa require accurate information on vector densities and species composition. Further, a clear understanding of the epidemiology of the disease is important if an effective intervention programme is to be developed. Monitoring of habitat productivity and vector population dynamics would provide critical information for vector surveillance in areas where control interventions are implemented. In sub-Saharan African countries, *Anopheles gambiae *sensu lato (s.l.) and *An*. *funestus *groups contain the most efficient malaria transmitting vectors [4-6].

In the recent past, western Kenya highlands have experienced increased frequencies of malaria epidemics and are now reported to have high rates of transmission [7-11]. Significant increases in the human population and subsequent land use changes, such as deforestation and swamp cultivation, have been hypothesized to be among the several mechanisms responsible for this increase in malaria epidemics in the highland regions of western Kenya, [12,13]. Swamp reclamation for agricultural development has resulted in the creation of potential breeding habitats for *An*. *gambiae *s.s and *An. arabiensis *[4,14]. High rates of deforestation have led to a rise in local temperatures which has been shown to lead to increased mosquito larvae survivorship and development of parasites in adult mosquitoes [13,15]. These factors have increased the productivity of malaria vectors [13], thus increasing the risk of malaria transmission at these highland sites [10].

The principal malaria vector *An*. *gambiae *s.l has been demonstrated to breed in sunlit, temporary water bodies such as hoof prints, goldmines, and drainage ditches while *An*. *funestus *is typically associated with permanent water bodies such as swamps and drainage ditches [4,16]. The habitats of these vectors in these highlands are highly concentrated in valley bottoms in both the rainy and dry seasons [17,18]. Although adult emergence from habitats is the primary determinant of vector density, the productivity of larval habitats for aquatic stages of the malaria vector *Anopheles gambiae *(the stage preceding adult metamorphosis) is not clearly understood [19]. This is partly contributed to by methodological problems associated with larval ecology studies of *Anopheles gambiae *[20]. For example, at the moment, there is no standardized method for accurate estimation of productivity of adult mosquitoes from larval habitats. However, this is essential for effective vector surveillance and planning larval control programmes [21]. Thus, there is need to evaluate an efficient and sensitive method for estimating malaria vector productivity in different habitats so as to translate the information into meaningful epidemiological data for monitoring *An*. *gambiae *s.l. and *An*. *funestus*. Accurate information on habitat productivity can lead to the determination of the seasons during which habitat source reduction targeting is appropriate, thus leading to vector reduction [22,23]. Source reduction have shown to be effective in vector reduction in different part of Africa [24-27]

In estimating habitat productivity, three main methods have been used for sampling different mosquito larvae: dipping, netting, and pipetting [21]. These techniques have been used to estimate the larval density in different habitat types. Among them, pipetting is predominately used in estimating the productivity of tree holes in breeding mosquitoes [21]. Use of netting and dipping methods however are limited in habitats with higher grass cover [21]. Due to the inconsistency of these methods, there is need to evaluate emergence traps and aerial samplers in estimating habitat productivity in different seasons and habitats to determine if they would be more effective than methods used previously. Therefore, the objective of this study was to compare the efficiency of the emergence trap and the aerial sampler in estimating larval habitat productivity under different habitat types in highland areas of western Kenya.

## Materials and methods

### Study area

**Figure 1:**
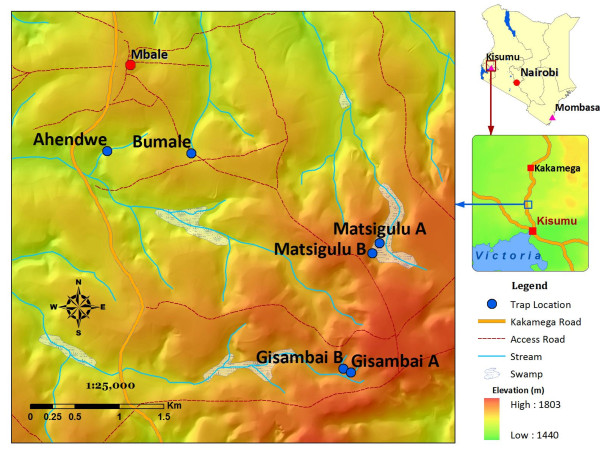
**A map showing the study sites in western Kenya highlands**.

The present study was conducted in six sites at Mbale village, Vihiga district in the western Kenyan highlands (Figure 1). The area has different land use types and topography that have enhanced the availability of potential mosquito breeding habitats. The three main land use types at Mbale are farmland, pasture, and forests. No productive mosquito larval habitats were found in forested areas during the study period. Habitat types used were drainage ditches (these are canals constructed to channel excess water to nearby streams and rivers, from farmland), natural swamps, and abandoned goldmines. Drainage ditches were found in the farmland, while swamps and abandoned goldmines were found in pastures. The main cultivated crops are tea, maize, beans, finger millet, and sorghum.

### Larval habitats selection and survey

The different types of larval habitats, i.e., swamps, drainage ditches, and abandoned goldmines, were identified at the beginning of the study in each season. For each survey, the different types of breeding sites were sampled using standard dippers (350 ml). Ten dips were made at each habitat and the presence or absence of mosquito larvae was recorded. In the first rainy season survey, (June - 2008 to August 2008), sixty habitats were selected and each habitat type had ten emergence traps and ten aerial samplers. In the first, (February-2009 to April 2009) and the second dry season (February-2010 to April, 2010), and the second rainy season (June-2009 to August, 2009), 66 habitats were sampled using eleven paired emergence traps and aerial samplers for each habitat type per season. Each trapping method was done during dry and wet season in two different years to avoid yearly weather change effects.

**Figure 2:**
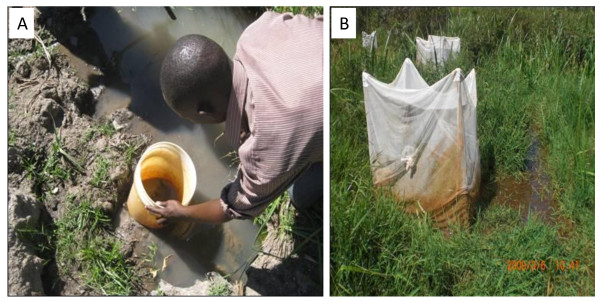
**(A) The aerial sampler used for estimation of habitat productivity by field technician; (B) The emergence trap positioned in habitat for productivity estimation**. **Consent**: The person shown in the photograph has consented to publication.

The aerial samplers used in this study had a diameter of 0.28 m and depth of 0.30 m, and were used eight times per habitat (surface area of 0.5 m^2^) in each sampling day. (Figure 2A). The emergence trap was made of an iron-framed cage, either 1 m × 0.5 m × 1 m (surface area of 0.5 m^2^) or 1 m × 1 m × 1 m (surface area of 1 m^2^), covered by fine mosquito polystyrene netting material (Figure 2B). The 0.5 m^2 ^traps were used for small goldmines and drainage ditches while 1 m^2 ^traps were used for large goldmines and swamps. Emergence traps were shifted daily in the same habitat to allow gravid mosquitoes to lay eggs. In all seasons, the samplings were done for 60 days consecutively for both emergence traps and aerial samplers.

### Mosquito sampling and processing

Adult mosquitoes were sampled from emergence traps using a mechanical aspirator as described in the World Health Organization (WHO) entomological field manual [21]. Pupae sampled in the aerial sampler were retained in a field insectary until the adults emerged. All adults were anaesthetized using chloroform, and identified using a morphological key in the laboratory as described in Gillies and Coetzee [28]. Mosquito specimens were preserved in absolute ethanol (purity 96%) for molecular species identification using the polymerized chain reaction (PCR) protocols developed by Scott *et al*., [29] for *An*. *gambiae *s.l. and Koekemoer *et al.,*[30] for *An*. *funestus *group. We utilized a morphological key developed by Darsie *et al. *[31] for culicine species identification.

### Data analysis

Data were recorded daily by date, sampling method, habitat type, and mosquito species. Data analysis was performed using PWAS Statistics 18 (SPSS Inc., Chicago, IL). The most efficient sampling method for estimating mosquito habitat productivity was chosen by comparing sampling methods in all seasons using paired sample t-tests. Assessment of the effects of seasonality, habitat type, and sampling method in estimating habitat productivity were analysed by multi-factorial analysis of variance (MANOVA) using species as a dependent variable. Pairwise comparison of mosquito productivity with method, habitat type, and season was done using Tukey's HSD test of MANOVA with repeated measure. The contribution of algal cover, grass cover, habitat depth and width, and habitat water surface area on species productivity was analysed using stepwise regression analysis (only culicines species, *An*. *gambiae s*.*l*. and *An*. *funestus *were included; other species were excluded due to their low densities). The efficiency of the sampling methods in estimating habitat productivity was determined using the three most abundant species (culicine, *An*. *gambiae *s.l. and *An*. *funestus*) for each season, using cumulative habitat productivity.

## Results

A total of 11,514 female mosquitoes were sampled during the study period. Out of these, 5,014 (43.5%) were *An*. *gambiae *s.l., 1,247 (10.8%) *An*. *funestus *and 5,253 (45.6%) were culicine species mosquito. Drainage ditches produced 4,565 (39.6%), abandoned goldmines 2,887 (25.1%), and swamps 4,062 (35.3%) mosquitoes. Among ten percent (501) of all *An. gambiae *s.l. identified; 303 (60.5%) were identified as *An*. *gambiae *s.s, 169 (33.7%) were *An*. *arabiensis *and 29 (5.8%) specimens of mosquitoes had no PCR products. Among identified *An*. *gambiae *s.s, 152 (50.2%) were sampled from drainage ditches while 151 (49.8%) were from abandoned goldmines. For *An*. *arabiensis, *109 (64.7%) came from drainage ditches and 60 (35.3%) were from abandoned goldmines.

The 10% (525) of all *An*. *funestus *group identified, 514 (97.9%) were found to be *An*. *funestus *while the 11 (2.1%) mosquitoes specimens had no PCR products. All *An*. *funestus *were sampled, came from swamps. By using a morphological key, it was revealed that all culicines were *Culex quinquefasciatus*.

Overall estimation of habitat productivity by comparison of sampling method has shown the aerial sampler to be the more efficient method in estimating the productivity of anopheline species while emergence traps did better for culicine species (Table 1). Upon comparison, it was found that for *An*. *gambiae *s.l. and *An*. *funestus*, the aerial sampler performed better by 21.5 and 14.6 folds than the emergence trap respectively. However, the two sampling methods showed no difference in estimating the productivity of *An*. *implexus *(Table 1).

**Table 1:** Performance of emergence trap and aerial sampler in estimating habitat productivity (female/m^2^) for each mosquito species sampled.

Species	Aerial sampler (Mean ± SE)	Emergence trap (Mean ± SE)	Paired t-test *P*-value
*Anopheles gambiae *s.l.	706.30 ± 39.36	32.80 ± 27.79	0.024
*Anopheles funestus*	36.66 ± 4.25	2.51 ± 0.35	< 0.001
*Anopheles squamous*	238.77 ± 27.46	1.43 ± 0.24	< 0.001
*Anopheles coustani*	13.25 ± 2.19	0.51 ± 0.10	< 0.001
*Anopheles ziemann*	4.31 ± 1.13	0.15 ± 0.05	< 0.001
*Anopheles implexus*	3.67 ± 1.06	3.39 ± 0.99	0.196
*Culex *species	63.36 ± 18.79	113.88 ± 2.99	0.008

The pairwise analysis using Tukey's HSD test of MANOVA with repeated measures showed that, the productivity of *An*. *gambiae *s.l. was significantly higher in drainage ditches than in any other habitat types for all seasons. *An*. *funestus *productivity in swamps was significantly higher than in other habitat types throughout all seasons (Tukey HSD test *P *< 0.05) (Table 2). The culicine species productivity was significantly higher in goldmines when compared with habitat types and sampling methods in all the seasons (Tukey HSD test *P *< 0.05).

**Table 2:** Mosquito productivity estimated by different sampling methods in different season and habitat types

Sampling method	Sampling season		Productivity (female/m^2^)		
			
		Habitat type	*An. gambiae*	*An. funestus*	Culicine
Area sampler	June-August, 2008	Drainage ditch	2.28	0.97	0.11
		Gold mine	1.08	0.17	4.66
		Swamp	1.56	0.76	0.65
	
	June-August, 2009	Drainage ditch	1.82	0.03	0.03
		Gold mine	0.28	0.02	0.15
		Swamp	0.03	0.76	0.20
	
	February-March, 2009	Drainage ditch	40.48	0.39	0.34
		Gold mine	20.41	0.30	0.00
		Swamp	16.93	0.03	0.13
	
	February-April, 2010	Drainage ditch	0.90	0.19	0.26
		Gold mine	1.15	0.05	0.29
		Swamp	0.14	0.64	0.43

Emergence trap	June-August, 2008	Drainage ditch	0.35	0.01	0.32
		Gold mine	0.07	0.00	0.37
		Swamp	0.05	0.01	1.05
	
	June-August, 2009	Drainage ditch	0.53	0.00	1.36
		Gold mine	0.20	0.00	0.51
		Swamp	0.25	0.04	1.59
	
	February-March, 2009	Drainage ditch	1.17	0.08	1.48
		Gold mine	0.17	0.00	1.43
		Swamp	0.36	0.13	3.03
	
	February-April, 2010	Drainage ditch	0.20	0.00	0.59
		Gold mine	0.25	0.00	0.81
		Swamp	0.31	0.11	1.04

Results of stepwise regression analysis show that the productivity of *An*. *gambiae *s.l was significantly associated with high algae cover (*P *< 0.0001), low grass cover (*P *< 0.0001) and debris (*P *= 0.05), but chlorophyll a, habitat width and depth had no significant effect. Only grass coverage (*P *< 0.01) showed a significant effect on the production of *An*. *funestus*. In assessing the impact of environmental factors on productivity of culicine species, only chlorophyll a (*P *= 0.05) was found to have a significant effect.

**Figure 3:**
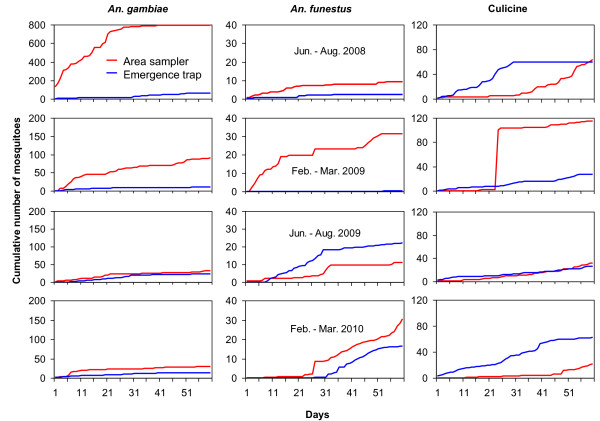
**The efficiency of estimating *An. gambiae *s.l. (left), *An. funestus *(middle) and *Culicine *(right) species productivity by emergence trap and aerial sampler in all four seasons (shown in different rows)**.

The efficiency of the sampling methods was found to vary in all four seasons for all three mosquito species. The aerial sampler was shown to be a better tool than emergence traps for estimating habitat productivity in both dry and rainy seasons (Table 2, Figure 3).

## Discussion

The findings of this study indicate that aerial sampler is the most efficient in estimating habitat productivity of malaria vectors in different habitat types and seasons in the highlands of western Kenya. Mosquito species identified from *An. gambiae *s.l and *An*. *funestus *group have revealed the malaria vectors found in this study area are *An. gambiae *s.s, *An. arabiensis *and *An*. *funestus*. *An*. *gambiae *s.s and *An*. *funestus *have been found to be anthropophilic vectors while *An*. *arabiensis *is both anthropophilic and zoophilic depend on geographical location and host availability [28,32]. Similar species composition information was reported from other sites in the western Kenyan highlands [6,33], where mosquitoes were sampled by other methods. In this study mosquito species productivity was found to be habitat-type dependent as *An*. *gambiae *s.s and *An. arabiensis *were abundant in drainage ditches and abandoned goldmines, *An*. *funestus *in swamps, and Culicine species in abandoned goldmines. This pattern was similar to other studies conducted in western Kenya [13,14,33,34].

Aerial samplers performed better than emergence traps in estimating the productivity in larval habitats, presumably due to the netting material covering the emergence trap which inhibited gravid mosquitoes from laying eggs in those habitats. Aerial samplers showed higher efficiency for all species' productivity estimations and did not disrupt mosquito oviposition in habitats, thereby producing more reliable estimates [21]. Aerial samplers yielded more reliable information on mosquito larval habitat productivity in the study areas for control purposes than other methods in previous studies [35,36]. Unlike other studies conducted in western Kenya [13,19,35,36], in all 60 days of every season, aquatic habitat stability had no effect on the estimation of the productivity because habitats remained stable throughout although the size of the habitats varied with the seasons. The sample size used indicates that the efficiency of these sampling methods in estimating productivity can be useful where any of these species are found. These methods can be utilised in areas of Africa where malaria vectors breeding habitats are prevalent and have increased as a result of environmental degradation and land use changes [17,36,37]. The efficiency of these methods provides additional information for planning improved larval habitat interventions intending to reduce the adult density effectively [38]. Larval habitat control by use of larvicides has been a major practice in urban malaria control programmes in Africa [25-27,39], but the use of these methods evaluated here may significantly reduce the costs of larvicides and increase the efficacy of targeted larval habitat control programmes. Previously used methods for estimating habitats productivity such as dipping, netting, and mobile and static quadrant devices, were not effective in terms time spent in sampling and operational costs as evaluated traps [21,40]. Belleville traps were structurally better but were not useful as the methods evaluated in this study due to their lower ability to estimate the productivity in habitats with deep water, higher grass cover, water turbidity, algal abundance and debris cover which could not affect current methods [40]. The reported methods in this study are more reliable in estimating vectors population in an area of interest for vector intervention than other methods such as human landing catch, pyrethrum spray catch, pit trap, and odour-baited traps [21,41-43]. The failure of the above mentioned methods in estimating local vector densities seasonally is based on the fact that they estimate only adult densities that might have flown from other locations for host-seeking. A study conducted in West Africa showed aerial samplers and emergence traps to be efficient in estimating the abundance of anopheline and culicine mosquito species during the rainy season in river beds and rice fields [44].

Seasonality differences were observed to have contributed small changes in estimating of the vector density. These traps' efficiency in estimating habitat productivity was mostly affected by other natural factors such as dry and rainy seasons' variations. In Figure 3, both trapping methods used had lower density estimation efficiency for all species in second dry season (February 2010 - April 2010) than in the first dry season (February 2009 - March 2009). This might be explained as the effect of over washing which was attributed to El Nino (heavy) rains which took place between November, 2009 and January, 2010. The habitats where larval productivity was most affected were the drainage ditches. These ditches are small in size and therefore, are more susceptibility to instability during long periods of drought or flooding during heavy rains. Since *An. gambiae *ss and *An. arabiensis *were found predominantly in these ditches, their productivity was affected by the habitat flooding. *An*. *funestus *and culicine species productivity estimation were higher as swamps and abandoned goldmines had larger volume of water that could support their gravid female oviposition sites longer than the drainage ditches. It is probable that the heavy rains experienced during the rainy season from June to August 2009 and subsequently El Niño rains affected the productivity of *An*. *gambiae *s.s, *An. arabiensis *and culicine species as drainage ditches and abandoned goldmines were flooded and washed out respectively. Meanwhile *An*. *funestus *productivity was not affected due to the large size of the swamps and their ability to retain large amounts of water (Figure 3). In the rainy season from June to August 2008, rainfalls were short and less intense and therefore, facilitated better productivity of *An*. *gambiae *s.l in drainage ditches as there was no flooding of ditches resulting in more oviposition sites for the gravid Anopheles females. Productivity of culicine species in this season was also favoured as no flooding in the goldmines leading to increased larvae survivorship and subsequently a higher density of pupae and adults (Figure 3). For *An*. *funestus*, the productivity was found to decrease in swamps during this rainy season with the shorter rains due to the fact that the shorter rains could not reduce the amount of brown and green algal cover in habitats and hence the larval abundance. The algal cover favoured culicine species abundance at high levels of decompositions and *An. gambiae *s.l abundance at lower levels of decomposition.

Despite the efficiency of the aerial sampler, other habitat ecological factors such as chlorophyll a, debris abundance, grass cover and algae abundance were found to influence productivity of mosquitoes. *An*. *gambiae *ss and *An. arabiensis *were found to have significant relationship with presence of debris, low grass cover and algal abundance. In other studies it was found that, *An*. *gambiae *s.l larvae use plant debris and algal biomass as a food source thus increasing productivity of the habitats [13,45]. Lower grass coverage implies that these habitats are exposed to more sunlight. Previously it has been shown that larval habitats in open areas are more likely to have higher water temperatures, resulting into acceleration of the development rate, increased survival of *An. gambiae *s.l larvae and decreased larval developmental time into adults [13,45]. *An*. *funestus *was found to occur in large water bodies significantly associated with high grass coverage as reported in previously ecological studies [28,46]. The increase of chlorophyll-a has been associated with increased habitat pollution, a phenomenon that favoured culicine species larvae resulting in higher densities in polluted habitats [13,37,46,47].

Studies done on mosquito dynamics in the highlands of western Kenya in the past, showed that *An. gambiae *s.s and *An. funestus *were the main malaria vectors [18,48,49]. Previous studies reported *An. arabiensis *abundance at 1.3% from larval habitats and 3% from indoor spray catch respectively [13,24]. Another study by Wamae and colleagues [50] reported *An. arabiensis *abundance of 10% in the highlands. This study is the first one of its kind to determine *An. arabiensis *abundance of 33.7% among all the mosquitoes specimens identified by PCR sampled for two years. This can be justified in three ways; first, this high occurrence of *An. arabiensis *in habitats has been in contrary to the number of adults collected indoors by pyrethrum spray catches (PSC). The low number of *An. arabiensis *caught by PSC can be attributed to the duality of the *An. arabiensis *behaviour in that it is zoophagic (i.e. feeding on animals), exophilic (i.e. prefers to rest outdoors) and exophagic (i.e. prefers feeding outdoors) [28,32,51]. Secondly, the high rate of bednet ownership and usage reported among community members in this area might have contributed to the low indoor mosquito densities [52,53]. Thirdly, environmental degradation (including deforestation) and land use type changes might have contributed to the creation of convenient habitats and raised temperatures for *An. arabiensis *[54,55]. This study has also revealed that *An. arabiensis *has reinvaded and as begun establishing itself within the Kenyan highlands due to high abundance observed ever reported.

The aerial sampler information reported here has the potential to reduce the cost of planning for targeted intervention of malaria vectors species as the preferred habitats of each species is already known. This method allow for improved temporal and spatial planning for the monitoring and control of malaria transmission and epidemics in the highlands of western Kenya and in other highlands and urban areas of Africa. Currently, there are major malaria control efforts underway in Africa, and these have demonstrated remarkable success in malaria vectors reduction in selected countries [56] where these methods can be of additional value in empowering malaria vector larval habitats control programmes.

## Conclusion

These results have given additional information in understanding of habitat productivity for targeted intervention and control of anopheline and culicine species in highland and urban areas of Africa. These sampling methods have been proven to have potential for improved efficiency in the planning for integrated vector management.

## Conflict of interest

All authors declare they have no competing interest in this study.

## Authors' contributions

EJK, AKG, SM and GY conceived, designed and implemented the study; EJK, TMG and SM conducted field experiments. AKG and GY did the supervision of the project activities. EJK, ML and GZ did data analysis and interpretation. EJK wrote this manuscript. AKG, GZ, FM, EJK and GY revised the manuscript. All authors edited and approved the final version.
